# Clinical forms of peritoneal larval cestodiasis by *Mesocestoides* spp. in dogs: diagnosis, treatment and long term follow-up

**DOI:** 10.1007/s00436-021-07107-w

**Published:** 2021-03-09

**Authors:** Silvia Carta, Andrea Corda, Claudia Tamponi, Giorgia Dessì, Francesca Nonnis, Laura Tilocca, Agostina Cotza, Stephane Knoll, Antonio Varcasia, Antonio Scala

**Affiliations:** 1 Laboratorio di Parassitologia, Ospedale Didattico Veterinario, Dipartimento di Medicina Veterinaria, Università degli Studi di Sassari, Via Vienna, 2 –, 07100 Sassari, Italy; 2 Centro Veterinario Roma Sud, Roma, Italy

**Keywords:** CPLC, Diagnosis, Dogs, Mesocestoidosis, Tetrathyridium, Treatment

## Abstract

Canine peritoneal larval cestodiasis (CPLC) is a little-known parasitological infestation of the peritoneal cavity of wild and domestic carnivores with *Mesocestoides* spp. larvae. While adult *Mesocestoides* tapeworms reside within the small intestine, the larvae occasionally penetrate the host’s intestinal wall, causing a potentially life-threatening peritonitis. Severity of infection as well as the host response influences the prognosis significantly, and early diagnosis and treatment are essential. However, due to the lack of specific symptoms, this condition is underdiagnosed and, furthermore, no clear effective treatment has yet been described. The aim of this study is therefore to report two clinical cases of CPLC in dogs and to illustrate their clinical presentation and follow-up to serve as a reference for clinicians and researchers alike. Both animals were presented with abdominal distention as their main complaint. They underwent clinical examination, abdominal ultrasonography, abdominocentesis, and laparotomy followed by biochemical, cytological, parasitological, and molecular examination of the collected samples. After surgical lavage, the dogs received anthelmintic treatment with either fenbendazole (FBZ) or praziquantel (PZQ). Overall, timely and prolonged administration of high doses of FBZ seems to be the most effective treatment method. Irrespective, to date, no treatment capable of complete eradication of the infection and prevention of recurrence of disease has been found. In conclusion, further investigation into appropriate treatment plans as well as diagnostic development is needed.

**Supplementary Information:**

The online version contains supplementary material available at 10.1007/s00436-021-07107-w.

## Introduction

*Mesocestoides* tapeworms belong to the order Cyclophyllidea and have a worldwide occurrence. So far, seven species have been recorded in Europe (e.g., in Czech Republic, Slovak Republic, and Spain) where *Mesocestoides litteratus* and *Mesocestoides lineatus* have been described as the most widely distributed (Literák et al. 2004, 2006; Tenora 2005; Hrčkova et al. 2011; Zaleśny and Hildebrand 2012). This being said, several recent surveys have shown evidence of new genetic variants within this genus, thus requiring further investigation (Montalbano Di Filippo et al. 2018; Varcasia et al. 2018; Berrilli and Simbula 2020).

*Mesocestoides* spp. are considered parasites of wild and domestic carnivores although these tapeworms have occasionally being reported in birds and humans as well (Wirtherle et al. 2007). In fact, *Mesocestoides* spp. are considered zoonotic with at least 26 cases previously reported, in particular in Japan, Korea, China, Ruanda, and Greenland (Fuentes et al. 2003; Széll et al. 2015). Regardless, many aspects of the biology of these worms still remain unknown and to date, although hypothesizing a foodborne origin, no evidence for the route for human infection has been shown (Fuentes et al. 2003).

According to current knowledge, two intermediate hosts are required for the completion of the *Mesocestoides* life cycle (Papini et al. 2010). The first larval stage of these parasites probably develops in coprophagous arthropods in which oncospheres reach the hemocoel and progress further into the cysticercoid stage. The second larval stage known as tetrathyridium can be found in a great variety of hosts (e.g., rodents, amphibians, reptiles and birds) and develops after ingestion of first intermediate hosts (Loos-Frank 1991; McAllister et al. 1991; Literák et al. 2004). Definitive hosts acquire the infection by ingestion of intermediate hosts containing infectious tetrathyridium larvae which, ultimately, develop into mature tapeworms in the host’s intestine.

However, in rare cases, definitive hosts (dogs and cats) can serve as second intermediate hosts as tetrathyridium larvae penetrate the intestinal wall and invade the host’s peritoneal cavity leading to a condition known as canine peritoneal larval cestodiasis (CPLC) (Crosbie et al. 1998; Siles-Lucas and Hemphill 2002; Boyce et al. 2011). Various forms of metacestodes, which can be found floating in abdominal fluid and/or enclosed in small cysts, have been associated with this disease, for example, tetrathyridia with an inverted scolex and four well-developed suckers and so-called acephalic larvae without a scolex nor suckers (Wirtherle et al. 2007).

Many questions regarding the pathogenesis of CPLC remain, and it is still unknown if tetrathyridia and acephalic larvae found in the peritoneal cavity of definite hosts represent larvae that have migrated through the intestinal wall following ingestion of second intermediate hosts or if these represent an aberrant stage resulting from accidental ingestion of first intermediate hosts (Boyce et al. 2011). Besides, CPLC patients can harbor both intermediate *Mesocestoides* stages as well as adult tapeworms coincidently raising more questions in this regard (Toplu et al. 2004). Finally, the discovery of *Mesocestoides* spp. tetrathyridia in the pleural space of a dog by Petrescu et al. (2020) has put the hypothesized intestinal migration route into question.

The clinical representation of CPLC can range from asymptomatic to a severe clinical picture. Secondary to peritonitis, this disease is characterized by abdominal distension, lethargy, anorexia, vomiting, urinary incontinence, polyuria, and polydipsia (Crosbie et al. 1998; Wirtherle et al. 2007; Papini et al. 2010; Boyce et al. 2011; Yasur-Landau et al. 2019).

Overall, the severity of clinical signs has a significant influence on prognosis and survival of the animal (Boyce et al. 2011). The degree of ascites in particular is considered as a direct indicator of parasite mass or numbers (Boyce et al. 2011). Subsequently, early diagnosis and appropriate therapy are essential for recovery of affected animals (Boyce et al. 2011). Unfortunately, due to the lack of specific symptoms, this condition is likely underdiagnosed or the diagnosis delayed leading to a fatal outcome (Yasur-Landau et al. 2019).

The aim of this study is therefore to describe the clinical, ultrasonographic, parasitological, and molecular findings of two cases of CPLC in dogs and illustrate their clinical presentation and therapy response. By doing so, we hope to provide researchers with sufficient background to further investigate this still little-known disease and to offer clinicians a reliable reference for the recognition, diagnosis, treatment, and follow-up of CPLC cases they might encounter.

## Materials and methods

The two dogs mentioned in this research were examined and treated by trained veterinary physicians. Primary actions taken included physical examination, complete blood count (CBC), blood smear, serum biochemistry, abdominal ultrasonography (using a micro-convex 8 C-RS multifrequency transducer, 4–11 MHz), and echo-guided abdominocentesis (Portable Logiq® eVet ultrasound, General Electric Company Fairfield, CT, USA). Following, both dogs underwent laparotomy and surgical lavage with a warm sterile 0.90% NaCl solution (250 ml/kg). Pathology specimens were collected and submitted for chemical, cytological, histopathological, and parasitological examination and isolation. Once parasite samples were isolated and identified, molecular analysis was performed to confirm morphological diagnosis. DNA was extracted using the commercial kit PureLink® Genomic DNA Mini Kit (Invitrogen, Carlsbad, California, USA) according to the manufacturer’s instructions, and polymerase chain reaction (PCR) was performed to amplify the partial fragments of the parasite’s mitochondrial cox1 and nad1 genes following previously described protocols (Littlewood et al. 2008; Hrčkova et al. 2011; Otranto et al. 2013; Varcasia et al. 2018). PCR products were purified using a Nucleospin Gel and PCR Clean Up (Macherey-Nagel GmbH & Co. KG, Düren, North Rhine-Westphalia, Germany) and sent to an external sequencing service (Eurofins Genomics, Germany). Finally, obtained sequences were compared with those found in the NCBI database using BLAST (http://www.ncbi.nlm.nih.gov/BLAST/).

## Results

Two dogs (from this point, case 1 and case 2) were presented for veterinary care after exhibiting abdominal distension, weakness, and anorexia.

### Case 1

A 16-year old, crossbreed, neutered male, 30 kg dog (Table 1) was presented to the Veterinary Teaching Hospital of the University of Sassari (Italy) for clinical examination. The dog was lethargic and tachypneic and with fever (39.7 °C). Mucous membranes were found to be congested and the abdomen distended and painful with palpation. Further results, including those of the CBC and biochemical analyses, can be found in Table 1.

**Table 1:** Clinical, hematological, and biochemical findings when dogs were first presented

Case	Animal	Anamnesis and clinical findings	Complete blood count	Biochemical analysis
1	Dog, 16 years old, neutered male, crossbreed, 30 kg	Abdominal distension Weakness Anorexia Lethargy Tachypnea Fever (39.7° C) Mucous membranes congested Abdomen distended and painful with palpation	Mild non-regenerative anemia (RBC 5 10^6^/μl) Leukocytosis (WBC 2810^3^/μl). Blood smear: neutrophilic leukocytosis with band and toxic neutrophils	Moderate increased alkaline phosphatase (350 mg/dl) Moderate increased alanine aminotransferase (200 UI/l) Hypoalbuminemia (Alb 2 g/dl)
2	Dog, 11-year-old, female, mixed breed, 29 kg	Abdominal distension Weakness Anorexia Polydipsia Vomiting Weight loss Tachypnea Tachycardia	Moderate anemia (RBC 5.2 × 106/μl) with band neutrophilia (6%) and without leukocytosis	Increase in blood urea Moderate hypoproteinemia (protein 6.0 g/dl) Hypoalbuminemia (2.1 g/dl) Moderate hypercalcemia High amylase (1350 UI/l)

Abdominal ultrasonography showed the presence of severe abdominal effusion characterized by echogenic and particulate fluid containing several rounded to cylindrical anechoic, cystic structures measuring 1–3 mm in diameter. Structures were found to be free-floating in the abdominal fluid or adherent to the serous surfaces, which appeared roughened and irregular. Mesenteric fat appeared hyperechoic with a coarse and irregular echostructure (Supplementary material: video).

Chemical and cytological examination of the abdominal fluid revealed the presence of an inflammatory exudate with high cellularity and protein content. A suspected diagnosis of chronic exudative peritonitis of parasitic origin was made.

**Fig 1:**
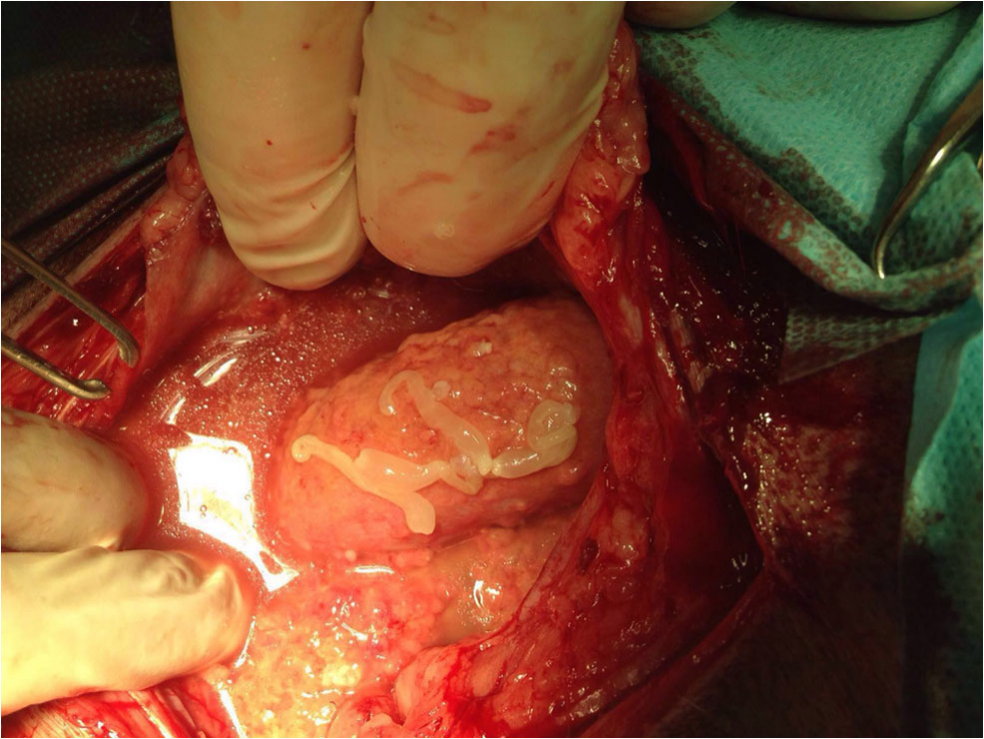
Abdominal cavity filled with sero-hemorrhagic fluid containing several free cystic and ribbon-like structures (case 1; Dr. Vittorio Tilocca)

**Fig 2:**
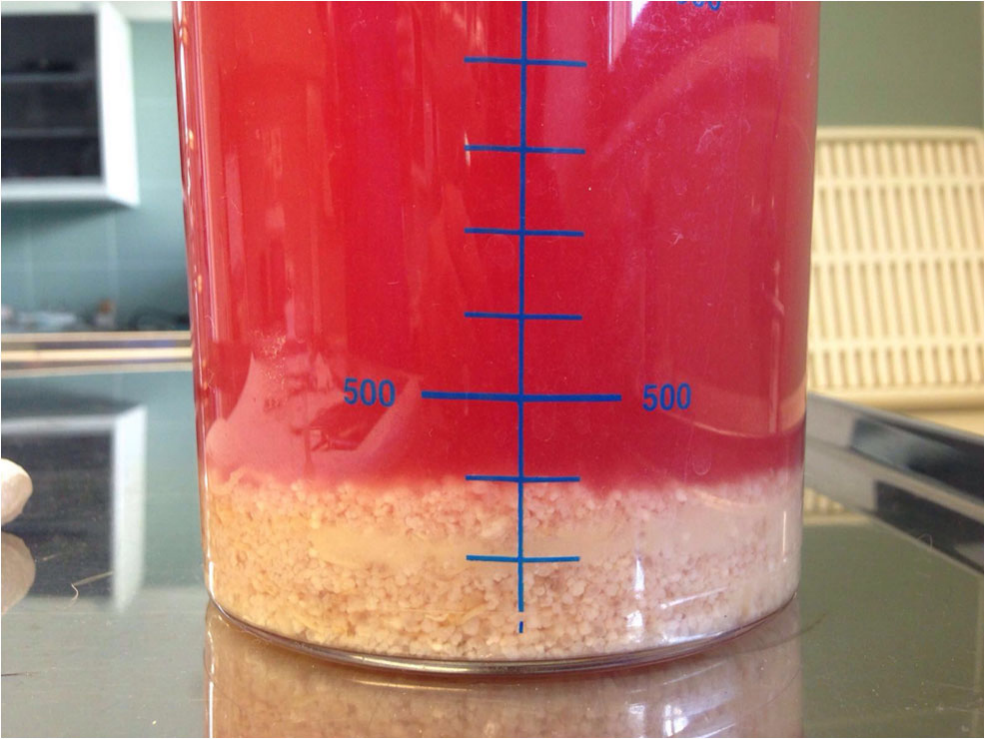
Sero-hemorrhagic fluid from abdominal cavity containing whitish particulated material (case 1; Dr. Vittorio Tilocca)

Exploratory laparotomy confirmed the presence of a severe peritonitis (Fig. 1). The abdominal cavity contained approximately 2 l of sero-hemorrhagic fluid with several free cystic and ribbon-like structures (Figs. 1 and 2). On microscopical examination, these elements were morphologically consistent with acephalic tetrathyridia, most likely belonging to *Mesocestoides* spp.

Molecular analysis of cox1 and nad1 partial mitochondrial gene fragments showed a homology of 99% with *Mesocestoides* spp. sequences found in GenBank (accession numbers MH463503 and MH463528).

The dog was discharged with an oral antibiotic [enrofloxacin 5 mg/kg (Baytril® tablets 150 mg, Bayer A.G. from Leverkusen, Germany), once a day for 10 days] and antiparasitic therapy [praziquantel, 10 mg/kg (Droncit® tablets 50 mg, Bayer A.G. from Leverkusen, Germany) orally administered, once a day for 4 days]. The dog showed a significant improvement and fully recovered 2 weeks after surgery.

Six months later, on re-examination, no hematological abnormalities were found and abdominal ultrasound did not reveal any free fluid or peritoneal cystic structures.

A year later, abdominal distention was observed again with pain elicited after abdominal palpation. Lethargy, inappetence, and tachypnea were also noted. Hematological results showed a moderate non regenerative anemia (RBC 4.3 × 10^6^/μl), thrombocytosis (599 × 10^3^/μl), and mild leukocytosis (WBC 17.6 × 10^3^/μl). Blood smear revealed the presence of band neutrophils and eosinophils. Biochemical profiling showed evidence of a moderate increase in alkaline phosphatase (390 mg/dl) and serum electrophoresis revealed hypoalbuminemia (1.8 g/dl) and hyperbetaglobulinemia (2.6 g/dl). Ultrasonography once more showed evidence of peritonitis with mild abdominal effusion and the presence of several anechoic cystic structures (1–3 mm in diameter) attached to the omentum. Ultrasonographic-guided collected abdominal fluid was compatible with inflammatory exudate. Cytological and molecular analysis confirmed *Mesocestoides* spp. infection recurrence.

The dog was hospitalized once more and underwent treatment with IV fluids (sterile 0.90% NaCl solution, 10 ml/kg), anti-inflammatory drugs [prednisone 1 mg/kg (Novosterol® Ceva Salute Animale Spa, Agrate Brianza, MB, Italy) intramuscularly, single dose], antibiotics [enrofloxacin 5 mg/kg (Baytril® tablets 150 mg, Bayer A.G. from Leverkusen, Germany) once a day for 10 days], and oral antiparasitic drugs [fenbendazole 50 mg/kg (Panacur® tablets 500 mg, MSD Animal Health Srl, Segrate, MI, Italy), twice a day for 28 days]. The patient improved clinically and was discharged from the hospital 3 days later.

Twenty days later, the dog was readmitted to the emergency clinic because of severe deterioration of its clinical condition. The dog was presented in acute shock, and all attempts to stabilize the animal were in vain. The dog passed away a few hours after admission. No consent for necropsy was given by the owner.

### Case 2

An 11-year-old, mixed breed, female, 29 kg dog (Table 1) was presented to the Clinica Veterinaria Roma Sud (Rome, Italy) for clinical examination. Besides the above mentioned symptoms, the dog had a history of polydipsia, vomiting, and tachypnea. The dog had been adopted from a kennel at 5 years of age and had been housed indoors with free access to an outside garden ever since. The animal had been regularly vaccinated and dewormed 2 years ago.

Physical examination revealed weight loss, tachycardia, tachypnea, and abdominal distension. Pathological laboratory findings are summarized in Table 1.

**Fig 3:**
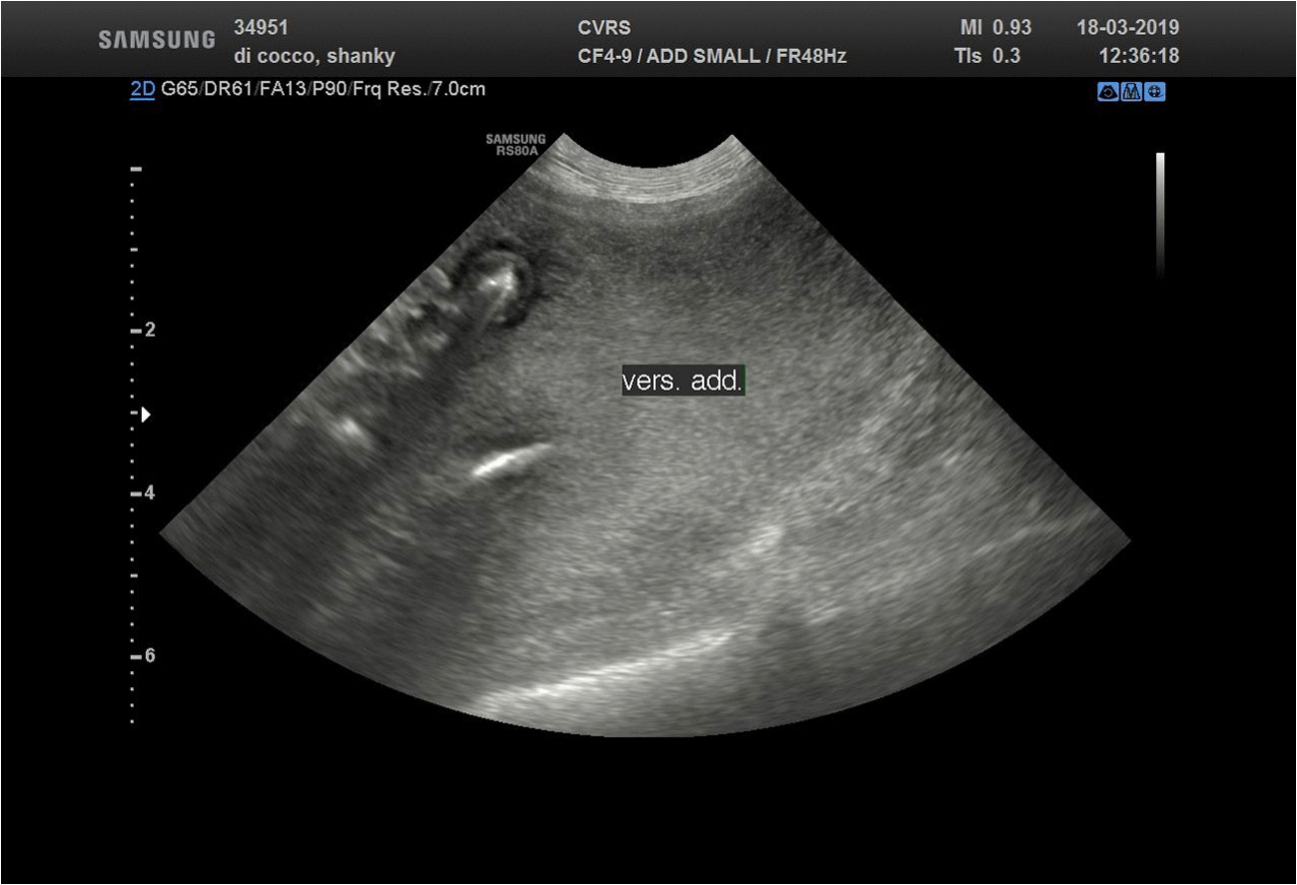
Abdominal ultrasonography showing severe abdominal ascites (case 2; Clinica Veterinaria Roma Sud)

Abdominal ultrasonography showed severe abdominal ascites (Fig. 3) with a hyperechogenic mesenterium and the presence of particulate fluid containing several rounded and anechoic cystic structures measuring 1–2 mm in diameter.

Abdominocentesis yielded a turbid whitish fluid classified as modified transudate/exudate with a specific gravity of 1026, a protein, triglyceride, and cholesterol content of 3.0 g/dl, 6 mg/dl, and 131 mg/dl respectively, and a high calcium concentration (25.2 mg/dl).

**Fig 4:**
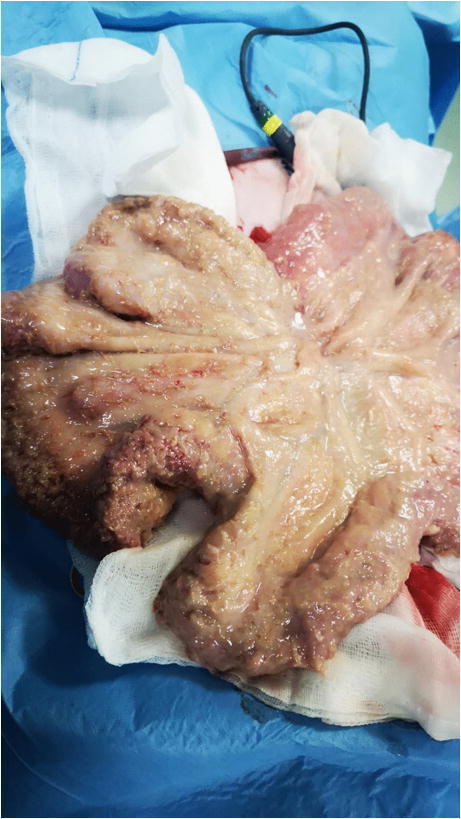
Peritoneal fibrinous adhesions on the visceral and parietal peritoneum (case 2; Clinica Veterinaria Roma Sud)

Exploratory laparotomy revealed peritoneal effusion and fibrinous adhesions on the visceral and parietal peritoneum (Fig. 4). Mesothelial hypertrophy, multifocal calcification, and amorphous structures were additionally noted. Moreover, the abdominal cavity was filled with numerous small, white cyst-like structures (0.5 to 4 mm) containing transparent liquid, partly attached to the serosa and partly floating.

**Fig 5:**
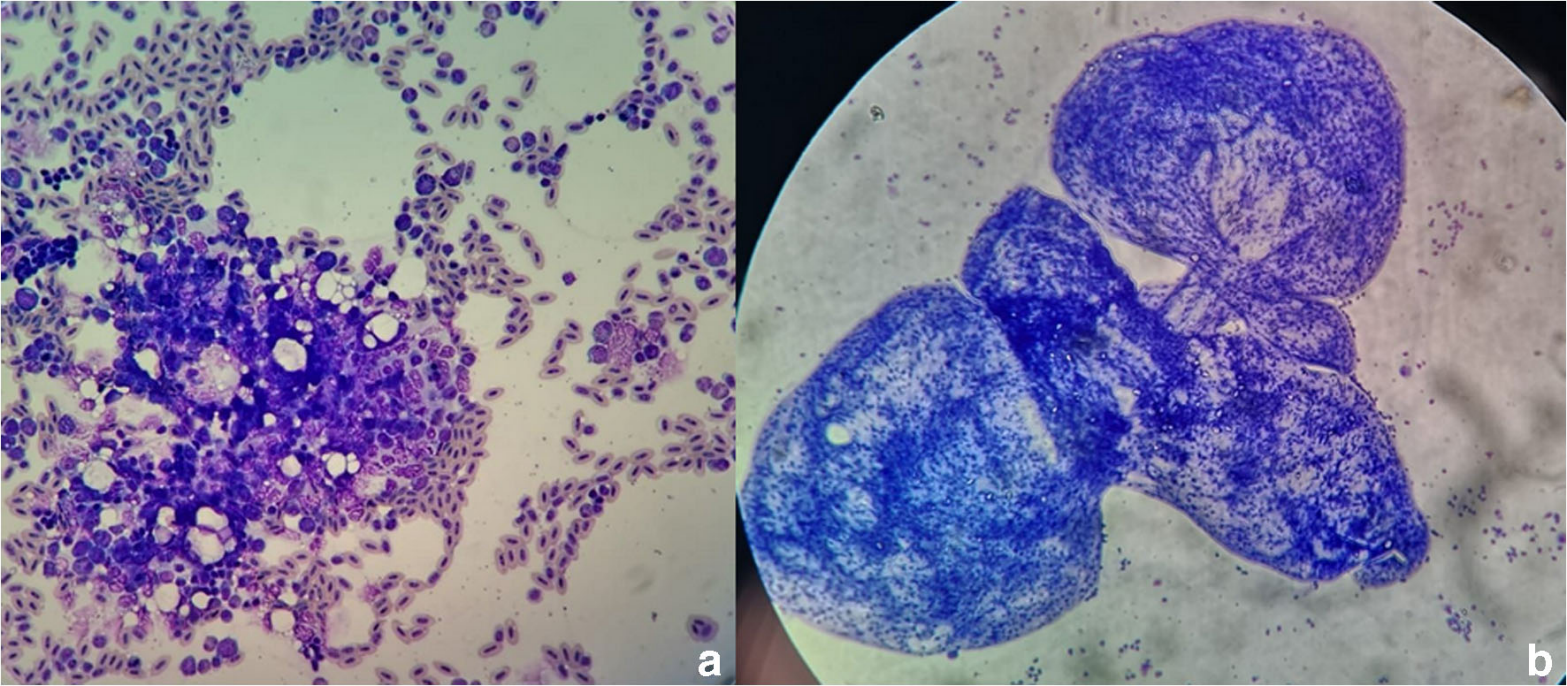
Granulomatous structures infiltrated by mononuclear cells (× 20) (**a**) and amorphous structures (× 2040) (**b**) from the abdominal cavity. Romanowsky stain (case 2; Clinica Veterinaria Roma Sud)

Cytological examination showed the cyst-like structures to be multifocal granulomatous, surrounded by a fibrous capsule and infiltrated by mononuclear cells (monocytes and macrophages) (Fig. 5). Within these structures, numerous whitish larval forms were observed and identified at microscopical examination as acephalic larvae filled with parenchyma and calcareous corpuscles. The larvae were morphologically identified as tetrathyridia belonging to *Mesocestoides* spp., and identification was confirmed through cox1 and nad1 sequencing (MH463493; MH463505; MH463519; MH463518).

Histopathology revealed the presence of chronic inflammatory lesions. Granulomatous structures infiltrated by plasma cells, macrophages, and eosinophilic granulocytes and nodular concentric structures with neutrophils, macrophages, and plasma cells, surrounded by a connective capsule were noted.

After 20 days of treatment with FBZ at 100 mg/kg (Panacur® tablets 500 mg, MSD Animal Health Srl, Segrate, MI, Italy) twice a day for 28 days, the dog’s condition improved substantially and no signs of peritonitis or abdominal effusion remained on ultrasonography. After a year of periodically repeated treatment regimens using the same drug and dosage, the patient appeared to have undergone full recovery.

Recently (December 2020), a recurrence of disease was noted that required renewal of oral FBZ treatment [Panacur® tablets 500 mg, MSD Animal Health Srl, Segrate, MI, Italy), 50 mg/kg twice a day for 28 days] to be implemented. At the time of submission of this paper, the dog remains in treatment.

## Discussion

Canine peritoneal larval cestodiasis is an uncommon, likely underdiagnosed condition and numerous questions still remain regarding the diagnosis and therapy of this disease. Furthermore, very little is known regarding CPLC as well as the biological aspects of *Mesocestoides* spp.

Following, although the pathogenesis of CPLC still remains unknown, both dogs in this research had free outdoor access and, therefore, exposure to potentially infected intermediate hosts, both first and second, is highly plausible and hence ingestion of infective tetrathyridia through this pathway remains the most likely scenario.

Overall, clinicians should be aware of CPLC as a differential diagnosis of peritonitis. This is of the utmost importance in order to recognize and treat this condition as soon as possible. Diagnostic procedures for animals affected by exudative peritonitis include ultrasonography, abdominocentesis, and, eventually, exploratory laparotomy (Wirtherle et al. 2007).

After said medical examinations, in both cases presented here, diagnosis of CPLC was made based on morphological characteristics of parasites found and confirmed through molecular analysis. Unfortunately, no serological tests exist for the detection of *Mesocestoides* spp., and fecal analysis is useless in the case of CPLC as this condition does not necessarily include the presence of adult tapeworms and, thus, often, no parasite stages are passed in the feces of the host (Tamponi et al. 2017). Besides, *Mesocestoides* spp. eggs can morphologically not be discriminated from other Taeniid species (Varcasia et al. 2004). Regardless, in case adult tapeworms would be present, the sensitivity of fecal flotation for the detection of *Mesocestoides* eggs has been found to be poor making this technique unreliable (Széll et al. 2015). Luckily, PCR analysis and sequencing of parasitic material represents a valuable tool for definite diagnosis and species differentiation of these tapeworms.

In Table 2, CPLC treatment protocols are reviewed as found in the most current scientific literature. Main protocols include administration of fenbendazole (FBZ) and praziquantel (PZQ) at different doses and frequency which, in most cases, reduce parasitic infestation and improve clinical condition but do not seem to prevent relapse.

**Table 2:** Treatment protocols for canine peritoneal larval cestodiasis (CPLC)

Drug	Dose (mg/kg)	Freq (h)	Route	Days	Follow-up	References
FBZ	50	24	PO	3 days/week	Animal died few weeks later from unknown causes	Crosbie et al. (1998)
FBZ	50	24	PO	28	Frequent recurrence of disease and larvae still present after few weeks	Crosbie et al. (1998)
FBZ	50	12	PO	14	Treatment discontinued because of bone marrow hypoplasia	Gary et al. (2004)
FBZ	50	12	PO	21	Larvae still present after treatments	Papini et al. (2010)
FBZ	100	12	PO	28–45	No recurrence of disease after 6–12 months Therapy was repeated in some cases	Crosbie et al. (1998); Caruso et al. (2003); Venco et al. (2005); Boyce et al. (2011); Yasur-Landau et al. (2019)
FBZ	50/100	24	PO	Life long	Dogs still in treatment at time of publication	Bonfanti et al. (2004); Boyce et al. (2011)
PZQ	5	12-13 (days)	SC	4	No improvements after 2 months and larvae still present	Yasur-Landau et al. (2019)
FBZ + PZQ	50 (FBZ) 5(PZQ)	24	PO (FBZ)	10 (FBZ) 1 (PZQ)	No recovery after treatment	Bonfanti et al. (2004)
FBZ + PZQ	50 (FBZ) 5(PZQ)	12(FBZ) 15 (days, PZQ)	PO (FBZ) SC (PZQ)	21 (FBZ) 2 (PZQ)	No larvae were observed after 14 months	Papini et al. (2010)
PZQ + IVM + FBZ	50/100 (FBZ)	12/24 (FBZ)	PO (FBZ)	28 (FBZ)	Larvae still present until FBZ was administered	Crosbie et al. (1998)
PZQ + MTZ + FBZ	50 (FBZ)	24 (FBZ)	PO (FBZ)	28+ 28 (FBZ)	No larvae present after FBZ was administered Therapy with FBZ was repeated	Crosbie et al. (1998)
PZQ + ABZ	50 (ABZ)	24 (ABZ)	PO (PZQ) IP (ABZ); SC (PZQ) PO (ABZ)	2 (ABZ) every 7days	No improvement	Crosbie et al. (1998)
PZQ + ABZ	25 (ABZ)	12 (ABZ)	PO (ABZ), PO or SC (PZQ)	14 (ABZ)	No improvement	Crosbie et al. (1998)

*FBZ* fenbendazole, *PZQ* praziquantel, *IVM* ivermectin, *ABZ* albendazole, *MTZ* metronidazole

PZQ is most commonly used to treat adult tapeworms in the small intestine and results of experimental studies have documented the effectiveness of this drug against *Mesocestoides* spp. tetrathyridia both in vitro (Saldaña et al. 2003; Markoski et al. 2006) and in vivo (Hrckova et al. 2007). This being said, administration of PZQ seems to have been ineffective against *Mesocestoides* spp. larvae residing within the peritoneal cavity of carnivorous hosts in previous reports (Boyce et al. 2011; Yasur-Landau et al. 2019). Irrespective, one case report did conclude PZQ to be more effective than FBZ in treating peritoneal cestodiasis (Papini et al. 2010) (Table 2).

In case 1, treatment with high doses of PZQ (administered orally) was effective in reducing the parasitic infestation and improves the clinical condition of the dog. However, this treatment was not sufficient for complete eradication of the infection and prevention of recurrence of disease.

In agreement with the findings of Papini et al. (2010), this paper shows repeated administration of PZQ to be effective in eliminating peritoneal tetrathyridia when provided before overt clinical signs are present. The authors do underline that this favorable outcome may have been influenced by the subcutaneous administration route of the drug. When orally administered, relatively small amounts of PZQ enter the systemic circulation (Badreldin 2006), while through subcutaneous route, the drug is absorbed directly into the systemic circulation, possibly resulting in greater therapeutic effectiveness against metacestodes in the peritoneal cavity (Papini et al. 2010). Similarly, the effectiveness of PZQ administration reported by Papini et al. (2010) might also have been biased due to the simultaneous administration of FBZ and PZQ. Furthermore, there are several other reasons to question the conclusions of said research, including the fact a small sample size was used (*n* = 1) and that persistent larvae may still have been present after treatment since the treatment efficacy was evaluated only by abdominal echography (Boyce et al. 2011).

The efficacy of simultaneous administration of both FBZ and PZQ against larval cestodes has been previously reported (Ghazaei 2007) although provision of a single dose of PZQ (5 mg/kg) in combination with oral FBZ (50 mg/kg, every 24 h, for 10 days) has been described to be ineffective in a 12-year-old mixed-breed male dog presenting CPLC (Bonfanti et al. 2004). Regardless, the authors did not provide any information regarding either the route or timing of the PZQ administration.

Treatment with high doses of FBZ seems to be most effective against CLPC and, as illustrated in Table 2, previous research has evidenced positive results against *Mesocestoides* spp. infection with this treatment (Crosbie et al. 1998). To this extent, since the most significant factors influencing survival of infected animals seem to be the severity of clinical signs at the time of diagnosis, application of immediate and aggressive treatment (FBZ 100 mg/kg twice daily for 28 days in combination with surgery/lavage) is recommended (Boyce et al. 2011). However, high-dose FBZ therapy is quite expensive and a single treatment cycle is often not effective in fully eradicating the larval infestation and preventing recurrence of disease (Crosbie et al. 1998; Bonfanti et al. 2004; Venco et al. 2005; Petrescu et al. 2020). Additionally, such treatment has been shown, in some cases, to be completely ineffective as well (Jura et al. 1998; Lucio-Forster et al. 2007). In case 2, treatment with high dose of FBZ seemed to have led to a full recovery of the animal when periodic treatment cycles were applied, but, also in this case, it was not sufficient to prevent disease recurrence.

Other protocols reported in Table 2 include treatment with PZQ either alone or in combination with other drugs (ivermectin, mebendazole, albendazole) as reviewed by Crosbie et al. (1998). However, dosage, route, and frequency of treatment were not currently reported (Papini et al. 2010). One dog was treated exclusively with oral PZQ, and three dogs were treated with PZQ orally, intraperitoneally, or both and in combination with albendazole per os. Applied treatments in these cases showed high toxicity, and no signs of clinical improvement were recorded. Ultimately, the larval infestation was not overcome leading to the untimely death of the animals.

Although showing evidence of the efficacy of prolonged, cyclic repetition of high-dose FBZ treatment after timely surgical intervention, the results of this study suggests that further research is needed in order to identify the most effective therapy against mesocestoidosis as well as the development of early diagnostic and therapeutic measures to prevent clinical manifestations.

## Conclusions

Canine peritoneal larval cestodiasis is a potentially life-threatening condition which should best be managed by early and prolonged treatment. Although PZQ and FBZ treatment may result in a rapid resolution of clinical manifestations and a reduction of parasitic infestation, they are not fully effective in eradicating the infection. This study emphasizes the need for further investigation into CLPC treatment development in order to identify the most effective therapy against mesocestoidosis. Furthermore, the authors suggest continued development and application of early diagnostics to be crucial in preventing severe clinical manifestations and thus for the probability of recovery from disease. Besides, taking into consideration the possible zoonotic potential of *Mesocestoides*, advancement in these fields could prove notably beneficial.

## Supplementary Information

ESM 1 Abdominal effusion containing several rounded and cylindrical anechoic, fluid-filled cystic structures free-floating in the abdominal fluid or adherent to the serous surfaces. (Case 1; Dott. Andrea Corda). (MP4 3845 kb)

## Data Availability

Not applicable
